# Rous Sarcoma Virus RNA Stability Element Inhibits Deadenylation of mRNAs with Long 3′UTRs

**DOI:** 10.3390/v9080204

**Published:** 2017-08-01

**Authors:** Vidya Balagopal, Karen L. Beemon

**Affiliations:** Biology Department, Johns Hopkins University, Baltimore, MD 21218, USA; vbalago1@jhu.edu

**Keywords:** Rous sarcoma virus, RNA stability element, nonsense mediated decay, long 3′UTR, deadenylation, decapping

## Abstract

All retroviruses use their full-length primary transcript as the major mRNA for Group-specific antigen (Gag) capsid proteins. This results in a long 3′ untranslated region (UTR) downstream of the termination codon. In the case of Rous sarcoma virus (RSV), there is a 7 kb 3′UTR downstream of the *gag* terminator, containing the *pol*, *env*, and *src* genes. mRNAs containing long 3′UTRs, like those with premature termination codons, are frequently recognized by the cellular nonsense-mediated mRNA decay (NMD) machinery and targeted for degradation. To prevent this, RSV has evolved an RNA stability element (RSE) in the RNA immediately downstream of the *gag* termination codon. This 400-nt RNA sequence stabilizes premature termination codons (PTCs) in *gag*. It also stabilizes globin mRNAs with long 3′UTRs, when placed downstream of the termination codon. It is not clear how the RSE stabilizes the mRNA and prevents decay. We show here that the presence of RSE inhibits deadenylation severely. In addition, the RSE also impairs decapping (DCP2) and 5′-3′ exonucleolytic (XRN1) function in knockdown experiments in human cells.

## 1. Introduction

### Retroviruses Have Aberrant mRNAs but Evade Cellular Nonsense-Mediated mRNA Decay

Retroviruses have compact genomes (<10 kilobases) that can code for eight or more viral proteins. In order to maximize the coding potential, major unspliced retroviral mRNAs often possess features such as long 3′ untranslated regions (UTRs), upstream open reading frames (uORFs), and retained introns that are predicted to be targets of host cell RNA surveillance machineries [1,2,3]. The unspliced RNA of Rous sarcoma virus (RSV), an avian retrovirus, carries a very long 3′UTR (ca. 7 kb); yet, it is very stable. A cis-acting RNA sequence found in the RSV genome, designated the RNA stability element (RSE; [4,5]) promotes viral evasion of the cellular nonsense-mediated mRNA decay NMD. The RSE, a 400-nt element located immediately downstream of the *gag* termination codon in the unspliced RSV viral RNA, has been shown to protect the viral RNA from Up-frameshift protein 1(UPF1) dependent decay in chicken cells [2,5,6,7,8]. Truncations in the RSE have defined a minimal RSE element of 155 nts [8].

More recently, the RSV RSE has been shown to protect cellular mRNAs with long 3′UTRs from NMD in mammalian cells [9]. Ge et al. inserted the RSE immediately downstream of the termination codon in a β-globin NMD reporter with a long SMG5 3′UTR, and found that it promoted stability of the reporter mRNA in human cells. Furthermore, the insertion of an antisense RSE fragment of similar length into this construct failed to stabilize it. The RSE RNA was shown to bind polypyrimidine tract binding protein 1 (PTBP1) and to decrease UPF1 association with the mRNA [9]. Interestingly, hundreds of cellular mRNAs with long 3′UTRs seem to have a similar PTBP1-binding sequence downstream of the termination codon that stabilizes the mRNAs [9]. However, the mechanism of mRNA stabilization by the RSE is unclear.

Degradation of NMD targets carrying premature termination codons (PTCs) has been shown to occur through exonucleolytic and endonucleolytic decay pathways [10,11]. mRNA decay is a multistep process regulated by several proteins. SMG6 has been shown to be important for endonucleolytic cleavage close to the PTC in some mRNAs [11]. Exonucleolytic degradation usually starts with the removal of the poly(A) tail (deadenylation). The deadenylated mRNA can then be decapped and subjected to 5′-3′ exonucleolytic decay. It can also become a substrate for the 3′-5′ exonucleases [10]. Accelerated deadenylation has been reported for PTC-containing NMD targets in mammalian cells [12,13]. However, the mechanism of decay of long 3′UTR-containing NMD targets is less well studied.

In this study, we asked how NMD targets containing long 3′UTRs are degraded and how the presence of the RSE allows them to evade NMD. We show that these NMD targets undergo deadenylation and decapping, as well as 5′-3′ exonucleolytic decay. Further, the presence of the RSE severely inhibits deadenylation and also impairs decapping and XRN1 mediated 5′-3′ exonucleolytic decay of the NMD reporter containing a long 3′UTR.

## 2. Materials and Methods

### 2.1. Cell Culture and Transfections

HeLa Tet-off Advance cells (Clonetech, Mountain View, CA, USA) were maintained in Dulbecco’s modified eagle medium (DMEM) supplemented with 10% fetal bovine serum, 1X antibiotic-antimycotic (Gibco 15240-062, Gaithersburg, MD, USA), and 0.3 mg/mL l-Glutamine (Life Technologies, Carlsbad, CA, USA). Cells were transfected with desired constructs using FUGENE 6 transfection reagent (Promega, Madison, WI, USA) according to the manufacturer’s protocol.

### 2.2. Analysis of Deadenylation and Decay

HeLa Tet-off Advance cells maintained in DMEM, supplemented with 10% fetal bovine serum (FBS) and 5 ng/mL doxycycline (Sigma, St. Louis, MO, USA), were seeded at a density of 1 million cells in 10-cm plates. For each plate, 4.8 μg of the indicated pcTET2-reporter plasmid was co-transfected with 1.2 μg of the wild-type β-globin reporter (βwt) control plasmid using FUGENE 6 transfection reagent (Promega) according to the manufacturer’s instructions. Twenty-four hours post-transfection, cells were split into six equal aliquots in six-well plates. The next day, cells were washed twice with 2 mL 1X Phosphate Buffered Saline (PBS) and incubated in medium without doxycycline for 5 h. Transcription was shut off by adding doxycycline to a final concentration of 1 µg/mL, and cells were harvested in Trizol (Life Technologies) after 30 min (time 0) and at the indicated intervals. Fifteen micrograms of the total RNA were annealed to oligonucleotide OVB117 (5′-GAAAGTGATGCTTTAGTCTCAGTC-3′) complementary to a sequence that is 214 nucleotides upstream of the poly(A) addition site of the reporter mRNA to generate a shortened RNA form to measure poly(A) tail length. RNaseH/oligo treatment of mRNA to generate poly(A) minus mRNA and measure deadenylation and decay were carried out as described in Reference [14]. Gel mobility of the RNA bands were measured using the scale tool on adobe Photoshop and sizes were determined by comparison of gel mobility to known size of the various bands of the end labelled ladder used. Amount of DNA at each time point was quantitated using densitometry and compared to a co-transfected control. mRNA levels from at least three biological replicates performed using extracts from cells transfected separately were used in each experiment.

To detect mRNA levels in cells depleted for specific factors by RNA interference (RNAi) HeLa Tet-off cells were depleted for the proteins using RNAi plasmids. HeLa tet-off cells were seeded at 0.2 million cells into 6-well plates and grown in DMEM supplemented with 10% FBS. After 24 h, each well was transfected with 500 ng of the indicated pcTET2 reporter plasmid and 500 ng of RNAi plasmid using Fugene 6 transfection reagent. Doxycycline was added to a final concentration of 5 ng/mL. Two days post-transfection, the wells were washed with 1X PBS reagent twice and supplemented with media without doxycycline to turn on transcription of the reporter mRNA. Cells were collected in Trizol after 12 h.

### 2.3. Northern Blotting

RNA was isolated using Trizol and resolved on 1.4% formaldehyde/agarose gels. ^32^P end-labeled oligonucleotides (GGAGTGGCACCTTCCAGGGTCAAG) against bovine growth hormone (bGH) polyadenylation signal present in both βwt and Tet-off reporter constructs were used for the detection of mRNAs. Northern blots were imaged on Typhoon phosphor imager scanners and quantification was performed using ImageQuant software (GE Healthcare Bio-Sciences Corp, Piscataway, NJ, USA).

## 3. Results

### 3.1. The RSV RSE Impairs mRNA Deadenylation

**Figure 1:**
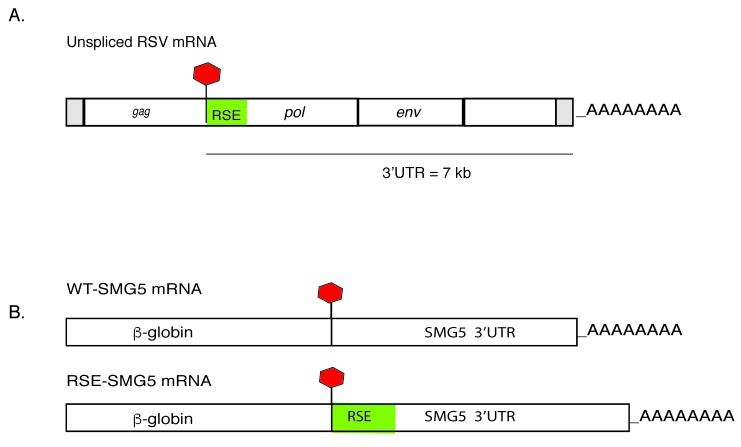
(**A**) Schematic of the Rous sarcoma virus (RSV) unspliced mRNA. The 400-nt RNA stability element (RSE) (shown in green) is located immediately downstream of the *gag* stop codon. This RNA has a very long 3′ untranslated region (UTR) of ca. 7 kb. (**B**) Schematic of tet-regulated β-globin reporter mRNA constructs used in our studies. WT-SMG5 mRNA contains the β-globin sequence followed by the SMG5 3′UTR sequence. The RSV RSE sequence was inserted into this reporter construct immediately after the β-globin stop codon to generate RSE-SMG5.

We have previously shown that the RSE can inhibit NMD of mRNAs with long 3′UTRs [5,9]. We wanted to understand how the RSE affects the mechanism of decay. Since deadenylation is often the first step in decay, we first studied the deadenylation rates of NMD reporters with long 3′UTRs in the presence or absence of the RSE. To address this question, we conducted a transcriptional-pulse chase experiment to determine the deadenylation and decay rates. Here, we used two reporter constructs, WT-SMG5 and RSE-SMG5, under the control of a Tet-off promoter ([9], Figure 1). The tetracycline (tet)-regulated reporter WT-SMG5 mRNA contains a β-globin mini-gene (with introns) and the SMG5 3′UTR. The SMG5 3′UTR has been shown to trigger NMD; this is proposed to be due to its length (1342 nt) [15,16,17]. The RSE-SMG5 construct carries a 400-nt RSE sequence inserted immediately downstream of the stop codon, mimicking the natural context of the RSE in the RSV RNA.

**Figure 2:**
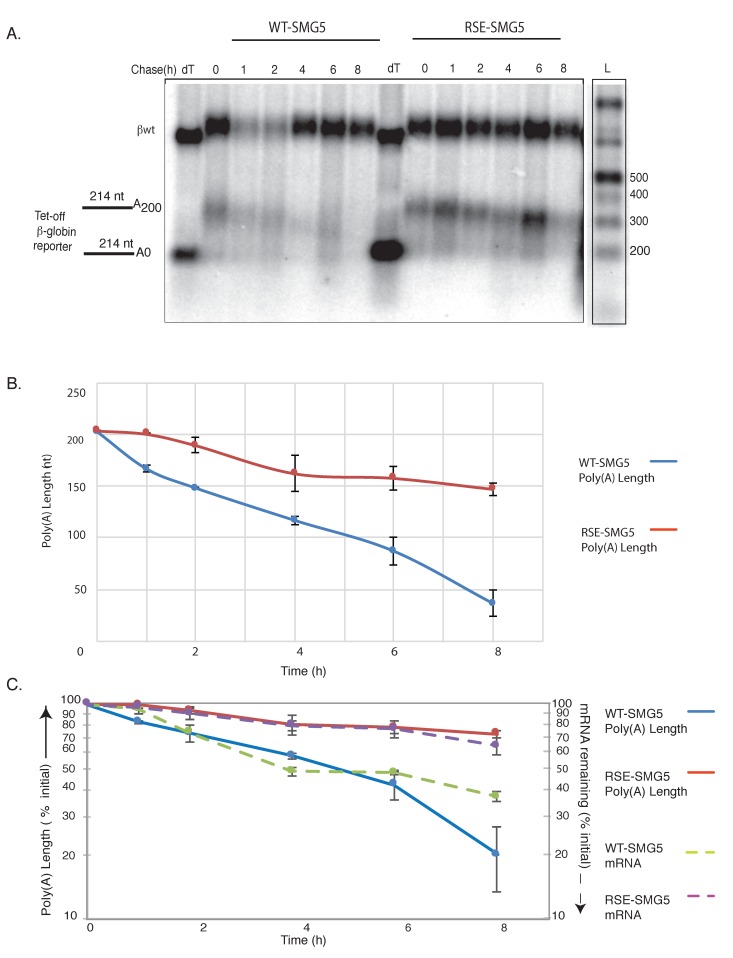
RNA stability element inhibits deadenylation. (**A**) Agarose northern blot showing deadenylation and decay of WT-SMG5 and RSE-SMG5 mRNAs following tet-off transcriptional pulse chase. RNA was subjected to oligonucleotide-directed RNaseH treatment prior to Northern blotting for tail length determination. Lanes marked “dT” show the length of the RNA fragment without a poly(A) tail; 1 kb plus DNA ladder (ThermoFisher Catalog number: 10787018, Halethorpe, MD, USA) was end labelled and used for size determination (size corrected for RNA). Wildtype β-globin (βwt) (upper band) was used as a loading control. (**B**) Poly(A) tail length determined from the gel was plotted against time to show the initial and final tail length of the two reporter constructs. The plotted values are the average of at least three separate experiments. Error bars show the standard error. Deadenylation rate of the reporters were also determined from this analysis. (**C**) Deadenylation and decay of the reporter mRNAs were compared by plotting mRNA remaining (% of original) and poly(A) length remaining (% of original tail length) on the same plot.

These reporter constructs were co-transfected into HeLa tet-off cells (Clonetech, Mountain View, CA, USA) with a vector constitutively expressing a control RNA. The expression of tet-regulated mRNAs was induced for 5 h before transcription was shut off by the addition of doxycycline, and mRNA was collected at the indicated time points. The mRNAs were then subjected to oligo-dT /RNaseH treatments followed by agarose Northern analysis to measure the poly(A) tail length and/or decay over the time course (Figure 2). At the starting time, both constructs generated mRNAs with poly(A) tails of approximately 200 nts (Figure 2B).

The WT-SMG5 reporter underwent rapid deadenylation at the rate of ca. 0.5 A/min, while the RSE-SMG5 reporter showed deadenylation rates that slowed down to ca. 0.1 A/min (Figure 2B). Deadenylation in mammalian cells usually proceeds in three steps. A slow initial deadenylation, usually by the weaker deadenylase complex Pan 2/3, results in ca. 110-nt A tails. This is followed by CCR4-NOT complex-mediated deadenylation resulting in a tail of ca. 22 As. In some cases, a terminal deadenylation occurs where all the As are removed [12]. We observed that the deadenylation of the RSE containing constructs did not surpass 70% of the original tail length in our 8-h time course. In contrast, the WT-SMG5 mRNA deadenylated to a poly(A) tail of <50 nts in 8 h (Figure 2B,C).

We compared the deadenylation and decay rates of WT-SMG5 and RSE-SMG5 reporters (Figure 2C). Transcripts containing only the SMG5 3′UTR exhibited a half-life of ca. 200 min while the transcripts containing the RSE were substantially more stable (half-life > 480 min), confirming the protective activity of the RSE in agreement with previous reports [9]. It is interesting to note that the decay rates of each reporter construct closely mirrored their deadenylation rates. This would suggest that the change in deadenylation is responsible for most of the alteration in decay.

### 3.2. Effects of the RSE on Decapping and 5′→3′ Exonucleolytic Decay

Nonsense mediated decay of mRNAs with long 3′UTRs could be exonucleolytic, initiating at either or both mRNA ends, or endonucleolytic. We assessed the role of the RSE in the regulation of decapping and 5′→3′ exonucleolytic decay. Decapping mRNA 2 (DCP2) or 5’-3’ Exoribonuclease 1 (XRN1) were depleted in HeLa-tet off cells using pSUPuro-Based RNAi plasmids previously characterized in Eberle et al. (generous gift from Oliver Muhlemann) [11]. Knockdown was performed by co-transfecting reporter constructs with pSUPuro-based RNAi plasmids against DCP2 or XRN1. We then measured the accumulation of the reporter constructs at steady state. As a control, RNAi against a scrambled sequence was separately carried out.

**Figure 3:**
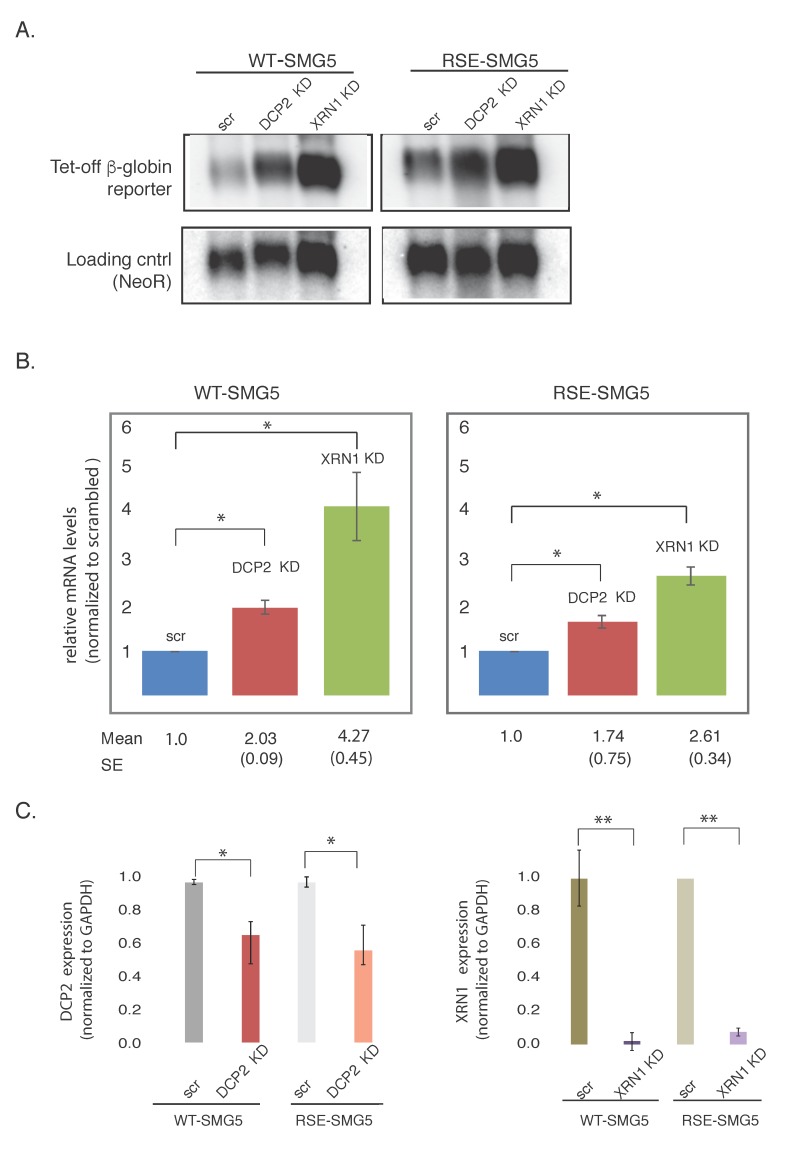
Depletion of decapping mRNA 2 (DCP2) or 5′-3′ Exoribonuclease 1(XRN1) leads to the accumulation of reporter mRNAs. (**A**) mRNA levels of the two reporters isolated from cells depleted for Exoribonuclease 1 (XRN1 KD), decapping mRNA 2 (DCP2 KD), or scrambled control (scr) were detected by agarose Northern blotting. NeoR, an mRNA produced from the same plasmid that generates the tet-off β-globin constructs was used as a loading control. Mean numbers show the amount of mRNA relative to scrambled control for WT-SMG5. SE shows the standard error of the values. (**B**) Left panel shows levels of WT-SMG5 reporter in different knockdown conditions normalized to the scrambled control. Right panel shows levels of RSE-SMG5 reporter in different knockdown conditions normalized to the corresponding scrambled control. The plotted values are the average of at least three separate experiments. Error bars show the standard error. (**C**) Quantitative reverse transcription polymerase chain reaction (RT-PCR) was carried out to determine the levels of knockdown of DCP*2* and XRN1. Expression levels of these mRNAs were measured relative to the housekeeping gene glyceraldehyde 3-phosphate dehydrogenase (GAPDH). Unpaired *t*-test was used to test for significance of the change in expression between each knockdown and the corresponding scrambled control. * denotes *p*-value < 0.05. ** *p*-value < 0.01.

Upon depletion of the major decapping enzyme DCP2, we found the WT-SMG5 reporter construct to be stabilized ca. 2-fold. The RSE-SMG5 construct was stabilized ca. 1.6-fold in this knockdown, as compared to the corresponding scrambled controls. Depletion of XRN1, the major 5′-3′ exonuclease, showed even greater accumulation of ca. 4.3-fold in WT-SMG5 and ca. 2.5-fold in RSE-SMG5 (Figure 3). Thus, impairment of decay by down regulating DCP2 or XRN1 had less effect on the RSE-containing construct than on the wildtype construct. This suggests the RSE is protecting the mRNA from degradation at the 5′ end of the mRNA as well as at the 3′ end. We cannot tell from these experiments whether the deadenylation precedes the decapping and 5′→3′ exonuclease activities.

We also noticed that XRN1 had a stronger effect on the accumulation of mRNA than DCP2 in both WT-SMG5 and RSE-SMG5 constructs. Knockdown of XRN1 mRNA appears to be more robust than DCP2 (Figure 3C). This could also be because there may be redundant decapping enzymes present in the cell such as NUDT16 [18,19]. We also observed that DCP2 and XRN1 depletions led to stabilizations of both reporter constructs. It is not surprising that the RSE-SMG5 reporter was stabilized upon depletion of both DCP2 and XRN1, as both proteins are components of the general decay pathway and are important for the decay of most normal messages. It should be noted that the WT-SMG5 constructs were always more stabilized than the RSE-SMG5 in both knockdowns.

It has been previously reported that the depletion of XRN1 leads to the accumulation of a 3′ mRNA fragment for NMD targets that is generated by endonucleolytic cleavage [11]. We were unable to detect the presence of 3′ fragments in our experiments. This would suggest that our NMD reporter does not undergo decay via endonucleolytic cleavage. It is, of course, possible that endonucleolytic cleavage is very sensitive to minor changes in conditions.

## 4. Discussion

mRNA degradation can be initiated by generating a new unprotected end vulnerable to attack by an exonuclease. In human cells, unprotected ends can be generated by endonucleolytic cleavage, deadenylation-dependent decapping, and exosome-mediated 3′-to-5′ decay. Deadenylation is one of the slowest and often rate-limiting steps in degradation. In this study, we looked at how the RSE protects long 3′UTR-containing mRNAs from NMD.

Our results show that the presence of the RSE severely inhibits deadenylation. We measured the tail length of the WT globin-SMG5 reporter to be ca. 200 As. The deadenylation rates of this NMD target mRNA was determined to be ca. 0.5 A/min. Both tail length and deadenylation rates are in agreement with previous studies for PTC-containing globin NMD reporters [14]. In the presence of the RSE, the WT-SMG5 reporter maintained the initial tail length of 200 As, but the deadenylation rate slowed down to approximated 0.1 A/min. This five-fold change in deadenylation is a significant impairment and could be a leading causal factor in the impairment of decay. It is also interesting to note that the deadenylation of the WT-SMG5 reporter proceeds to <50 As. The length of the poly(A) tail is known to affect translation and decay rates. mRNAs with short poly(A) tails (<50 A residues) are generally translationally repressed (reviewed in [20]).

We compared the deadenylation and decay rates to estimate the contribution of deadenylation to the degradation of the reporters. In both WT-SMG5 and RSE-SMG5, the decay rates follow the deadenylation rates closely. This is different from the case for the PTC-containing β-globin reporter, which undergoes rapid deadenylation but the decay rates seem to lag behind [13]. Our observation suggests that deadenylation plays an important role in the degradation of long 3′UTR-containing NMD targets and that the protective function of the RSE works through inhibiting deadenylation. How the RSE regulates deadenylation remains an interesting open question. We have previously shown that RSE can inhibit UPF1 binding to the RSE-SMG5 reporter mRNA. The UPF proteins, the SMG proteins, and the eRF1-eRF3 complex constitute the SURF complex that is important for triggering NMD in PTC-containing mRNA [21].

Nonsense mediated decay can be prevented by placing cytoplasmic poly(A) binding protein (PABP) in proximity to the termination codon, suggesting that the increased distance between the translation termination event and cytoplasmic PABP that results from termination at a PTC contributes to NMD [11,16,22,23,24]. UPF1 has also been shown to play an important role in accelerated decay in PTC-containing mRNAs [13]. This could be via interactions with SMG7 that has been shown to recruit the CCR4-NOT deadenylation complex by directly binding to POP2, its catalytic subunit [25]. The RSE could disrupt UPF1 binding, reducing SMG7 binding and hence the recruitment of the deadenylation complex. It has been reported that the degradation activity of SMG7 involves the decapping enzyme DCP2 and the 5′-to-3′ exonuclease XRN1 [25].

Using RNAi knockdown experiments, we show that the depletion of DCP2 and XRN1 results in the enhanced steady-state accumulation of mRNA with both reporter constructs. This is not surprising, as DCP2 and XRN1 are part of the general mRNA decay pathway. The effect of depletion of these factors is more pronounced in the WT-SMG5 NMD reporter mRNA. Since deadenylation, decapping, and XRN1 regulate the decay of the long 3′UTR-containing NMD target, we speculate that SMG7 might play an important role in the decay. Our results are in agreement with previous results with other NMD substrates. Lejeune and Maquat have shown the importance of decapping and 5′-3′ decay, as well as deadenylation, in the degradation of PTC-containing mRNA [10].

Given that poly(A) shortening is often the first and rate-limiting step in mRNA decay, viruses likely have developed cis and trans acting factors to repress or circumvent deadenylation [26]. Several families of RNA viruses, such as flaviviruses, bunyaviruses, and arenaviruses, have evolved 3′ terminal stem loop structures to stabilize the RNA in the absence of poly(A) tails [27]. Poliovirus infection promotes the degradation of poly(A) specific ribonuclease subunit 3 (PAN3), a protein that initiates the deadenylation of many cellular mRNAs [28]. Sindbis virus recruits the cellular HuR protein to stability elements in the 3′UTR of its transcripts to stabilize its poly(A) tail [29,30]. Kaposi’s sarcoma associated herpesvirus (KSHV) PAN RNA has a 79-nucleotide expression and nuclear retention element (ENE) that forms a triple helix structure with the poly(A) tail that inhibits degradation [31]. Our results using reporter constructs suggest that the Rous sarcoma virus RSE in its natural context functions to prevent NMD by inhibiting deadenylation, and thus adds to the growing number of examples of viruses manipulating deadenylation to escape cellular decay machinery.
